# 4-[(2-Phenyl­eth­yl)amino]­benzoic acid

**DOI:** 10.1107/S2414314624007533

**Published:** 2024-08-06

**Authors:** Kexin Zhang, Sihui Long

**Affiliations:** a School of Chemical Engineering and Pharmacy Wuhan Institute of Technology,Wuhan Hubei 430205 People’s Republic of China; University of Aberdeen, United Kingdom

**Keywords:** synthon, hydrogen bond, acid–acid dimer, crystal structure

## Abstract

The title compound, C_15_H_15_NO_2_, crystallizes with two mol­ecules in the asymmetric unit. In the crystal, the two mol­ecules associate to form an acid–acid dimer by pairwise O—H⋯O hydrogen bonds.

## Abstract 2

The title compound crystallizes with two mol­ecules in the asymmetric unit. In the crystal, the two mol­ecules associate to form an acid–acid dimer by pairwise O—H⋯O hydrogen bonds.

## Graphical abstract

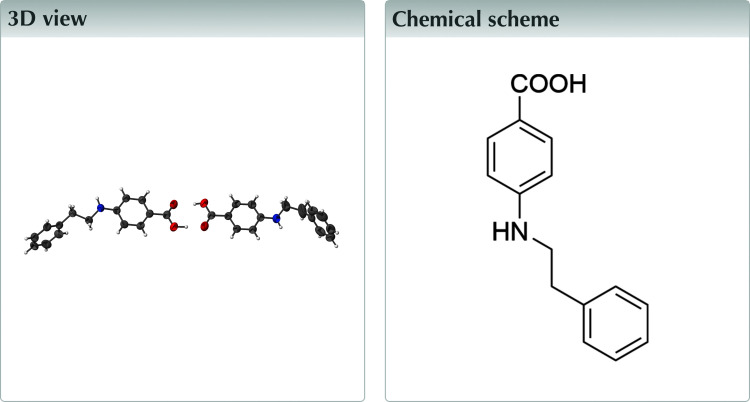

## Structure description

Non-steroidal anti-inflammatory drugs (NSAIDs) constitute approximately 5–10% of all prescribed medications worldwide as anti­pyretic, anti-inflammatory, and analgesic agents (Sohail *et al.*, 2023 ▸). As part of our ongoing studies in this area (Liu & Long, 2023 ▸), the title compound, C_15_H_15_NO_2_, was synthesized employing the Borch reductive amination reaction.

**Figure 1:**
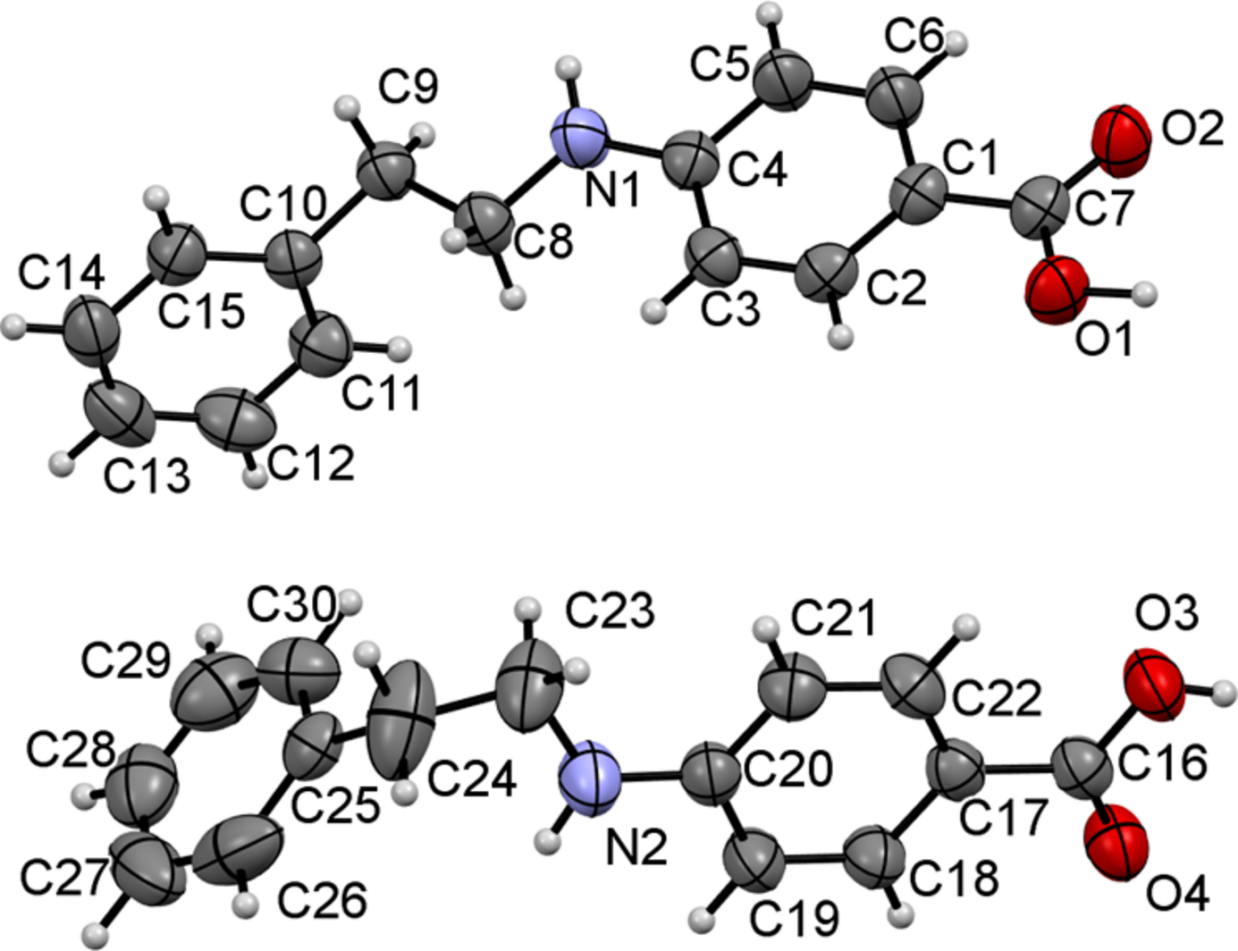
The mol­ecular structures of mol­ecules *A* and *B* in title compound with displacement ellipsoids drawn at the 50% probability level.

**Figure 2:**
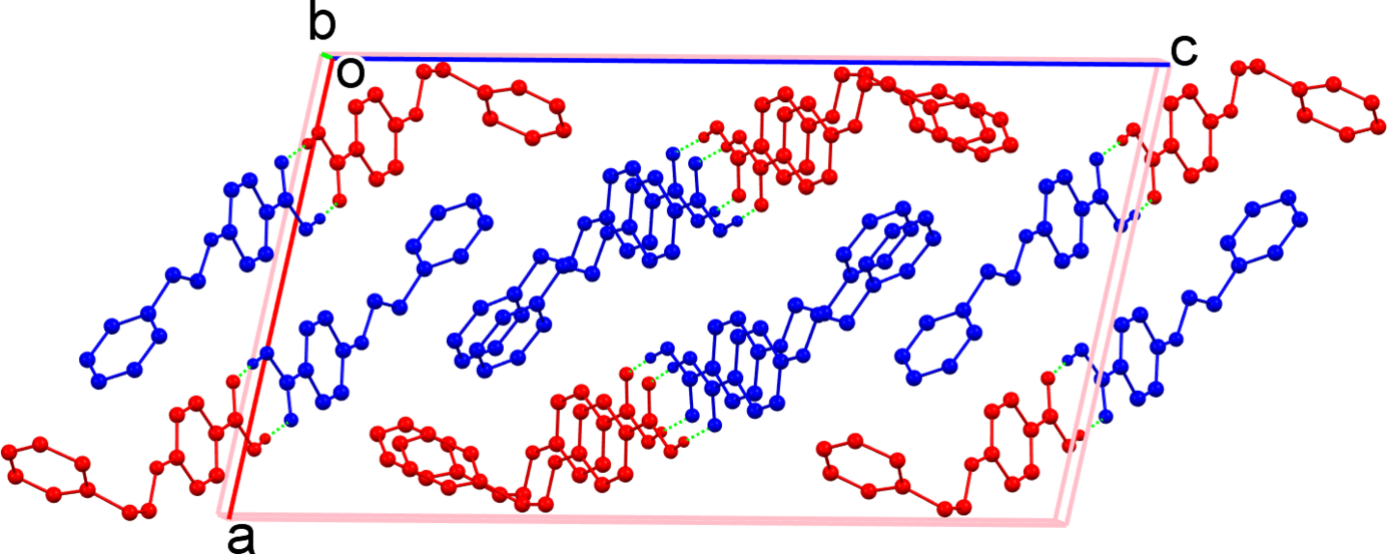
Packing of the mol­ecules in the title compound: C1 mol­ecule shown in blue, C16 mol­ecule shown in red (for clarity, H atoms not involved in hydrogen bonding are omitted).

There are two mol­ecules, *A* (containing C1) and *B* (containing C16), in the asymmetric unit (Fig. 1 ▸). Both mol­ecules are twisted with dihedral angles between their aromatic rings of 80.98 (9)° (mol­ecule *A*) and 83.54 (11)° (mol­ecule *B*). The main difference between the mol­ecules lies in the geometries of the linking ethyl-amino chains: the N1—C8—C9—C10 (mol­ecule *A*) torsion angle of 166.11 (15)° indicates an *anti* conformation whereas the N2—C23—C24—C25 (mol­ecule *B*) torsion angle of −59.3 (4)° is *gauche*. In the extended structure, the mol­ecules form *A*–*B* carb­oxy­lic acid dimers linked by pairs of O—H⋯O hydrogen bonds (Table 1 ▸, Fig. 2 ▸). Conversely, the NH groups do not participate in hydrogen bonds, presumably due to steric crowding.

## Synthesis and crystallization

**Figure 3:**
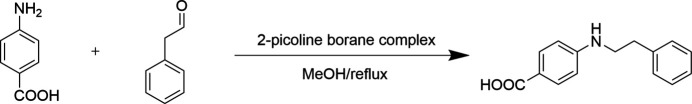
Synthesis scheme for the title compound.

The title compound was obtained by the reaction of 4-amino­benzoic acid and 2-phenyl­acetaldehyde using methanol as solvent in the presence of 2-picoline borane complex (Fig. 3 ▸). The crude product was recovered by filtration and purified by silica gel column chromatography. Colourless needles were produced by recrystallization from aceto­nitrile solution.

## Refinement

Crystal data, data collection and structure refinement details are summarized in Table 2 ▸.

## Supplementary Material

Crystal structure: contains datablock(s) global, I. DOI: 10.1107/S2414314624007533/hb4476sup1.cif

Structure factors: contains datablock(s) I. DOI: 10.1107/S2414314624007533/hb4476Isup2.hkl

Supporting information file. DOI: 10.1107/S2414314624007533/hb4476Isup3.cml

CCDC reference: 2374703

Additional supporting information:  crystallographic information; 3D view; checkCIF report

## Figures and Tables

**Table 1:** Hydrogen-bond geometry (Å, °)

*D*—H⋯*A*	*D*—H	H⋯*A*	*D*⋯*A*	*D*—H⋯*A*
O1—H1⋯O4	1.05 (4)	1.58 (4)	2.6380 (19)	177 (3)
O3—H3*A*⋯O2	0.82	1.81	2.6246 (18)	175

**Table 2:** Experimental details

Crystal data	
Chemical formula	C_15_H_15_NO_2_
*M*_r_	241.28
Crystal system, space group	Monoclinic, *P*2_1_/*c*
Temperature (K)	305
*a*, *b*, *c* (Å)	14.7698 (7), 6.6730 (3), 26.2392 (12)
β (°)	102.231 (5)
*V* (Å^3^)	2527.4 (2)
*Z*	8
Radiation type	Mo *K*α
μ (mm^−1^)	0.08
Crystal size (mm)	0.16 × 0.05 × 0.04

Data collection	
Diffractometer	XtaLAB Synergy R, DW system, HyPix
Absorption correction	Multi-scan (*CrysAlis PRO*; Rigaku OD, 2022 ▸)
*T*_min_, *T*_max_	0.598, 1.000
No. of measured, independent and observed [*I* > 2σ(*I*)] reflections	26873, 6498, 3944
*R*_int_	0.033
(sin θ/λ)_max_ (Å^−1^)	0.725

Refinement	
*R*[*F*^2^ > 2σ(*F*^2^)], *wR*(*F*^2^), *S*	0.058, 0.175, 1.03
No. of reflections	6498
No. of parameters	330
H-atom treatment	H atoms treated by a mixture of independent and constrained refinement
Δρ_max_, Δρ_min_ (e Å^−3^)	0.52, −0.28

Computer programs: *CrysAlis PRO* (Rigaku OD, 2022 ▸), *SHELXT* (Sheldrick, 2015*a* ▸), *SHELXL2018/3* (Sheldrick, 2015*b* ▸) and *OLEX2* (Dolomanov *et al.*, 2009 ▸).

## full crystallographic data

### 4-[(2-Phenylethyl)amino]benzoic acid Crystal data

**Table:**

C_15_H_15_NO_2_	*F*(000) = 1024
*M**_r_* = 241.28	*D*_x_ = 1.268 Mg m^−^^3^
Monoclinic, *P*2_1_/*c*	Mo *K*α radiation, λ = 0.71073 Å
*a* = 14.7698 (7) Å	Cell parameters from 6437 reflections
*b* = 6.6730 (3) Å	θ = 1.9–27.2°
*c* = 26.2392 (12) Å	µ = 0.08 mm^−^^1^
β = 102.231 (5)°	*T* = 305 K
*V* = 2527.4 (2) Å^3^	Needle, colourless
*Z* = 8	0.16 × 0.05 × 0.04 mm

### 4-[(2-Phenylethyl)amino]benzoic acid Data collection

**Table:**

XtaLAB Synergy R, DW system, HyPix diffractometer	6498 independent reflections
Radiation source: Rotating-anode X-ray tube, Rigaku (Mo) X-ray Source	3944 reflections with *I* > 2σ(*I*)
Mirror monochromator	*R*_int_ = 0.033
Detector resolution: 10.0000 pixels mm^-1^	θ_max_ = 31.0°, θ_min_ = 1.9°
ω scans	*h* = −21→19
Absorption correction: multi-scan (CrysAlisPro; Rigaku OD, 2022)	*k* = −8→9
*T*_min_ = 0.598, *T*_max_ = 1.000	*l* = −32→34
26873 measured reflections	

### 4-[(2-Phenylethyl)amino]benzoic acid Refinement

**Table:**

Refinement on *F*^2^	Primary atom site location: dual
Least-squares matrix: full	Secondary atom site location: difference Fourier map
*R*[*F*^2^ > 2σ(*F*^2^)] = 0.058	Hydrogen site location: mixed
*wR*(*F*^2^) = 0.175	H atoms treated by a mixture of independent and constrained refinement
*S* = 1.03	*w* = 1/[σ^2^(*F*_o_^2^) + (0.0816*P*)^2^ + 0.4926*P*] where *P* = (*F*_o_^2^ + 2*F*_c_^2^)/3
6498 reflections	(Δ/σ)_max_ = 0.001
330 parameters	Δρ_max_ = 0.52 e Å^−^^3^
0 restraints	Δρ_min_ = −0.28 e Å^−^^3^

### 4-[(2-Phenylethyl)amino]benzoic acid Special details

**Table:**

Geometry. All esds (except the esd in the dihedral angle between two l.s. planes) are estimated using the full covariance matrix. The cell esds are taken into account individually in the estimation of esds in distances, angles and torsion angles; correlations between esds in cell parameters are only used when they are defined by crystal symmetry. An approximate (isotropic) treatment of cell esds is used for estimating esds involving l.s. planes.
Refinement. The positions of H atoms attached to N1 and O1 were obtained from a difference Fourier map. Other H atoms were positioned geometrically with O—H = 0.82, N—H = 0.86 and C—H = 0.93 Å and constraioned to ride on their parent atoms with *U*_iso_(H) = 1.2*U*_eq_(carrier).

### 4-[(2-Phenylethyl)amino]benzoic acid Fractional atomic coordinates and isotropic or equivalent isotropic displacement parameters (Å^2^)

**Table:**

	*x*	*y*	*z*	*U*_iso_*/*U*_eq_	
O1	0.63928 (9)	0.5549 (2)	0.49996 (5)	0.0608 (4)	
H1	0.661 (2)	0.687 (5)	0.4841 (12)	0.139 (12)*	
O2	0.78373 (9)	0.5311 (2)	0.54712 (6)	0.0623 (4)	
N1	0.62345 (10)	−0.2690 (2)	0.62201 (6)	0.0556 (4)	
H1A	0.670942	−0.343326	0.633528	0.067*	
C1	0.67845 (12)	0.2741 (2)	0.55501 (6)	0.0448 (4)	
C2	0.58830 (12)	0.2008 (3)	0.54624 (7)	0.0475 (4)	
H2	0.540867	0.273251	0.525216	0.057*	
C3	0.56779 (12)	0.0226 (3)	0.56814 (7)	0.0473 (4)	
H3	0.506936	−0.022989	0.561997	0.057*	
C4	0.63833 (12)	−0.0896 (3)	0.59955 (7)	0.0461 (4)	
C5	0.72918 (12)	−0.0169 (3)	0.60742 (8)	0.0575 (5)	
H5	0.777273	−0.090155	0.627617	0.069*	
C6	0.74806 (13)	0.1609 (3)	0.58571 (8)	0.0539 (5)	
H6	0.808861	0.206790	0.591675	0.065*	
C7	0.70333 (13)	0.4639 (3)	0.53328 (7)	0.0485 (4)	
C8	0.53414 (12)	−0.3427 (3)	0.62782 (7)	0.0523 (4)	
H8A	0.496079	−0.374358	0.593891	0.063*	
H8B	0.502589	−0.241065	0.643982	0.063*	
C9	0.54902 (12)	−0.5296 (3)	0.66167 (7)	0.0496 (4)	
H9A	0.567642	−0.638343	0.641589	0.060*	
H9B	0.599654	−0.504918	0.691190	0.060*	
C10	0.46542 (11)	−0.5960 (2)	0.68191 (6)	0.0439 (4)	
C11	0.43655 (13)	−0.4860 (3)	0.72026 (7)	0.0554 (5)	
H11	0.466372	−0.366526	0.731813	0.067*	
C12	0.36428 (14)	−0.5512 (4)	0.74149 (8)	0.0678 (6)	
H12	0.345739	−0.476165	0.767353	0.081*	
C13	0.31917 (13)	−0.7279 (4)	0.72450 (9)	0.0671 (6)	
H13	0.270503	−0.772369	0.738992	0.080*	
C14	0.34607 (13)	−0.8363 (3)	0.68665 (8)	0.0629 (5)	
H14	0.315378	−0.954661	0.674913	0.075*	
C15	0.41898 (12)	−0.7717 (3)	0.66540 (7)	0.0525 (4)	
H15	0.437040	−0.847735	0.639567	0.063*	
O3	0.84499 (10)	0.8497 (2)	0.50638 (6)	0.0662 (4)	
H3A	0.822533	0.751596	0.517918	0.099*	
O4	0.69836 (10)	0.8856 (2)	0.46252 (6)	0.0632 (4)	
N2	0.87828 (12)	1.6586 (3)	0.38333 (7)	0.0675 (5)	
H2A	0.833024	1.729992	0.366770	0.081*	
C16	0.78021 (12)	0.9463 (3)	0.47444 (7)	0.0496 (4)	
C17	0.80772 (12)	1.1332 (3)	0.45255 (7)	0.0466 (4)	
C18	0.74220 (12)	1.2459 (3)	0.41908 (7)	0.0538 (4)	
H18	0.680858	1.203340	0.411462	0.065*	
C19	0.76571 (13)	1.4194 (3)	0.39684 (8)	0.0588 (5)	
H19	0.720120	1.493054	0.374767	0.071*	
C20	0.85731 (12)	1.4864 (3)	0.40705 (7)	0.0506 (4)	
C21	0.92346 (13)	1.3758 (3)	0.44124 (7)	0.0563 (5)	
H21	0.984766	1.418640	0.449118	0.068*	
C22	0.89844 (13)	1.2027 (3)	0.46351 (7)	0.0552 (5)	
H22	0.943452	1.130779	0.486442	0.066*	
C23	0.97058 (16)	1.7271 (4)	0.38434 (9)	0.0814 (7)	
H23A	1.001736	1.630511	0.366397	0.098*	
H23B	1.004391	1.734502	0.420262	0.098*	
C24	0.9728 (2)	1.9283 (5)	0.35931 (10)	0.1036 (10)	
H24A	1.037110	1.966035	0.362182	0.124*	
H24B	0.945471	2.025064	0.379169	0.124*	
C25	0.92401 (13)	1.9459 (3)	0.30261 (8)	0.0552 (5)	
C26	0.86614 (18)	2.1100 (4)	0.28705 (13)	0.0890 (9)	
H26	0.854239	2.202736	0.311259	0.107*	
C27	0.82450 (17)	2.1318 (4)	0.23115 (15)	0.0942 (9)	
H27	0.785947	2.239475	0.219054	0.113*	
C28	0.84354 (17)	1.9928 (5)	0.19821 (11)	0.0813 (7)	
H28	0.818089	2.005214	0.162771	0.098*	
C29	0.89698 (17)	1.8403 (5)	0.21468 (11)	0.0843 (7)	
H29	0.908093	1.745809	0.190688	0.101*	
C30	0.93649 (15)	1.8160 (3)	0.26544 (9)	0.0701 (6)	
H30	0.974023	1.704664	0.275262	0.084*	

### 4-[(2-Phenylethyl)amino]benzoic acid Atomic displacement parameters (Å^2^)

**Table:**

	*U*^11^	*U*^22^	*U*^33^	*U*^12^	*U*^13^	*U*^23^
O1	0.0613 (8)	0.0543 (8)	0.0630 (8)	0.0015 (6)	0.0047 (7)	0.0157 (6)
O2	0.0600 (8)	0.0528 (8)	0.0691 (9)	−0.0071 (6)	0.0024 (7)	0.0133 (6)
N1	0.0481 (8)	0.0523 (9)	0.0676 (10)	−0.0004 (7)	0.0151 (7)	0.0153 (7)
C1	0.0516 (9)	0.0429 (9)	0.0393 (9)	0.0004 (7)	0.0083 (7)	−0.0014 (7)
C2	0.0505 (10)	0.0479 (10)	0.0412 (9)	0.0034 (7)	0.0032 (7)	0.0007 (7)
C3	0.0458 (9)	0.0518 (10)	0.0441 (9)	−0.0039 (8)	0.0091 (7)	−0.0022 (7)
C4	0.0508 (9)	0.0451 (9)	0.0442 (9)	−0.0013 (7)	0.0138 (8)	−0.0006 (7)
C5	0.0459 (9)	0.0536 (11)	0.0690 (12)	0.0004 (8)	0.0035 (9)	0.0161 (9)
C6	0.0460 (9)	0.0509 (10)	0.0632 (12)	−0.0048 (8)	0.0080 (8)	0.0068 (8)
C7	0.0564 (10)	0.0440 (9)	0.0442 (9)	0.0007 (8)	0.0089 (8)	−0.0001 (7)
C8	0.0485 (9)	0.0545 (10)	0.0534 (10)	−0.0047 (8)	0.0099 (8)	0.0063 (8)
C9	0.0468 (9)	0.0494 (10)	0.0530 (10)	−0.0008 (8)	0.0113 (8)	0.0037 (8)
C10	0.0430 (8)	0.0460 (9)	0.0400 (8)	−0.0014 (7)	0.0030 (7)	0.0045 (7)
C11	0.0529 (10)	0.0597 (11)	0.0528 (10)	−0.0053 (9)	0.0092 (8)	−0.0099 (9)
C12	0.0603 (12)	0.0907 (16)	0.0558 (12)	0.0063 (11)	0.0201 (10)	−0.0033 (11)
C13	0.0450 (10)	0.0887 (16)	0.0674 (13)	−0.0044 (10)	0.0117 (9)	0.0236 (12)
C14	0.0538 (11)	0.0573 (12)	0.0734 (14)	−0.0133 (9)	0.0043 (10)	0.0092 (10)
C15	0.0545 (10)	0.0465 (10)	0.0542 (10)	−0.0002 (8)	0.0065 (8)	0.0004 (8)
O3	0.0646 (8)	0.0566 (8)	0.0739 (10)	0.0053 (6)	0.0068 (7)	0.0218 (7)
O4	0.0641 (8)	0.0550 (8)	0.0659 (9)	−0.0059 (6)	0.0033 (7)	0.0118 (6)
N2	0.0597 (10)	0.0657 (11)	0.0753 (12)	−0.0049 (8)	0.0104 (9)	0.0216 (9)
C16	0.0546 (10)	0.0474 (10)	0.0451 (9)	0.0041 (8)	0.0069 (8)	0.0023 (7)
C17	0.0517 (9)	0.0446 (9)	0.0431 (9)	0.0028 (7)	0.0093 (8)	0.0002 (7)
C18	0.0451 (9)	0.0550 (10)	0.0596 (11)	0.0006 (8)	0.0073 (8)	0.0095 (9)
C19	0.0509 (10)	0.0609 (11)	0.0626 (12)	0.0056 (9)	0.0076 (9)	0.0180 (9)
C20	0.0536 (10)	0.0489 (10)	0.0496 (10)	0.0004 (8)	0.0115 (8)	0.0030 (8)
C21	0.0477 (10)	0.0631 (12)	0.0549 (11)	−0.0058 (9)	0.0033 (8)	0.0017 (9)
C22	0.0539 (10)	0.0559 (11)	0.0509 (10)	0.0073 (8)	−0.0001 (8)	0.0056 (8)
C23	0.0672 (13)	0.1038 (19)	0.0657 (14)	−0.0280 (13)	−0.0031 (11)	0.0224 (13)
C24	0.126 (2)	0.114 (2)	0.0658 (15)	−0.0607 (19)	0.0075 (15)	0.0107 (15)
C25	0.0525 (10)	0.0566 (11)	0.0596 (11)	−0.0118 (9)	0.0191 (9)	0.0030 (9)
C26	0.0799 (15)	0.0647 (14)	0.139 (3)	−0.0150 (12)	0.0612 (17)	−0.0271 (15)
C27	0.0561 (13)	0.0780 (17)	0.145 (3)	0.0076 (12)	0.0123 (16)	0.0388 (18)
C28	0.0611 (13)	0.098 (2)	0.0812 (17)	−0.0161 (14)	0.0059 (12)	0.0191 (15)
C29	0.0666 (14)	0.108 (2)	0.0783 (17)	−0.0111 (14)	0.0154 (13)	−0.0114 (15)
C30	0.0577 (12)	0.0682 (13)	0.0840 (16)	0.0033 (10)	0.0139 (11)	−0.0099 (12)

### 4-[(2-Phenylethyl)amino]benzoic acid Geometric parameters (Å, º)

**Table:**

O1—H1	1.05 (4)	O3—H3A	0.8200
O1—C7	1.295 (2)	O3—C16	1.301 (2)
O2—C7	1.249 (2)	O4—C16	1.250 (2)
N1—H1A	0.8600	N2—H2A	0.8600
N1—C4	1.372 (2)	N2—C20	1.373 (2)
N1—C8	1.446 (2)	N2—C23	1.433 (3)
C1—C2	1.391 (2)	C16—C17	1.466 (2)
C1—C6	1.387 (2)	C17—C18	1.383 (2)
C1—C7	1.467 (2)	C17—C22	1.389 (3)
C2—H2	0.9300	C18—H18	0.9300
C2—C3	1.382 (2)	C18—C19	1.374 (3)
C3—H3	0.9300	C19—H19	0.9300
C3—C4	1.401 (2)	C19—C20	1.396 (3)
C4—C5	1.400 (2)	C20—C21	1.390 (3)
C5—H5	0.9300	C21—H21	0.9300
C5—C6	1.370 (3)	C21—C22	1.380 (3)
C6—H6	0.9300	C22—H22	0.9300
C8—H8A	0.9700	C23—H23A	0.9700
C8—H8B	0.9700	C23—H23B	0.9700
C8—C9	1.520 (2)	C23—C24	1.498 (4)
C9—H9A	0.9700	C24—H24A	0.9700
C9—H9B	0.9700	C24—H24B	0.9700
C9—C10	1.510 (2)	C24—C25	1.514 (3)
C10—C11	1.384 (3)	C25—C26	1.396 (3)
C10—C15	1.381 (2)	C25—C30	1.346 (3)
C11—H11	0.9300	C26—H26	0.9300
C11—C12	1.375 (3)	C26—C27	1.473 (4)
C12—H12	0.9300	C27—H27	0.9300
C12—C13	1.381 (3)	C27—C28	1.337 (4)
C13—H13	0.9300	C28—H28	0.9300
C13—C14	1.354 (3)	C28—C29	1.304 (4)
C14—H14	0.9300	C29—H29	0.9300
C14—C15	1.382 (3)	C29—C30	1.346 (3)
C15—H15	0.9300	C30—H30	0.9300
			
C7—O1—H1	114.6 (18)	C16—O3—H3A	109.5
C4—N1—H1A	117.5	C20—N2—H2A	117.8
C4—N1—C8	125.08 (15)	C20—N2—C23	124.32 (18)
C8—N1—H1A	117.5	C23—N2—H2A	117.8
C2—C1—C7	123.29 (16)	O3—C16—C17	116.54 (16)
C6—C1—C2	118.06 (16)	O4—C16—O3	122.73 (17)
C6—C1—C7	118.65 (16)	O4—C16—C17	120.73 (16)
C1—C2—H2	119.3	C18—C17—C16	119.79 (16)
C3—C2—C1	121.34 (16)	C18—C17—C22	117.64 (16)
C3—C2—H2	119.3	C22—C17—C16	122.56 (16)
C2—C3—H3	119.9	C17—C18—H18	119.3
C2—C3—C4	120.23 (16)	C19—C18—C17	121.49 (17)
C4—C3—H3	119.9	C19—C18—H18	119.3
N1—C4—C3	123.51 (16)	C18—C19—H19	119.7
N1—C4—C5	118.33 (16)	C18—C19—C20	120.67 (17)
C5—C4—C3	118.14 (16)	C20—C19—H19	119.7
C4—C5—H5	119.6	N2—C20—C19	118.91 (17)
C6—C5—C4	120.78 (17)	N2—C20—C21	122.79 (17)
C6—C5—H5	119.6	C21—C20—C19	118.30 (17)
C1—C6—H6	119.3	C20—C21—H21	119.9
C5—C6—C1	121.43 (17)	C22—C21—C20	120.20 (17)
C5—C6—H6	119.3	C22—C21—H21	119.9
O1—C7—C1	117.37 (16)	C17—C22—H22	119.2
O2—C7—O1	122.76 (17)	C21—C22—C17	121.67 (17)
O2—C7—C1	119.88 (16)	C21—C22—H22	119.2
N1—C8—H8A	110.0	N2—C23—H23A	109.0
N1—C8—H8B	110.0	N2—C23—H23B	109.0
N1—C8—C9	108.58 (15)	N2—C23—C24	112.8 (2)
H8A—C8—H8B	108.4	H23A—C23—H23B	107.8
C9—C8—H8A	110.0	C24—C23—H23A	109.0
C9—C8—H8B	110.0	C24—C23—H23B	109.0
C8—C9—H9A	108.6	C23—C24—H24A	108.1
C8—C9—H9B	108.6	C23—C24—H24B	108.1
H9A—C9—H9B	107.6	C23—C24—C25	116.8 (2)
C10—C9—C8	114.73 (14)	H24A—C24—H24B	107.3
C10—C9—H9A	108.6	C25—C24—H24A	108.1
C10—C9—H9B	108.6	C25—C24—H24B	108.1
C11—C10—C9	120.06 (16)	C26—C25—C24	118.9 (2)
C15—C10—C9	121.85 (17)	C30—C25—C24	123.5 (2)
C15—C10—C11	117.99 (17)	C30—C25—C26	117.5 (2)
C10—C11—H11	119.6	C25—C26—H26	121.0
C12—C11—C10	120.79 (19)	C25—C26—C27	118.0 (2)
C12—C11—H11	119.6	C27—C26—H26	121.0
C11—C12—H12	119.9	C26—C27—H27	120.8
C11—C12—C13	120.1 (2)	C28—C27—C26	118.3 (2)
C13—C12—H12	119.9	C28—C27—H27	120.8
C12—C13—H13	120.1	C27—C28—H28	119.3
C14—C13—C12	119.75 (19)	C29—C28—C27	121.5 (3)
C14—C13—H13	120.1	C29—C28—H28	119.3
C13—C14—H14	119.9	C28—C29—H29	119.1
C13—C14—C15	120.25 (19)	C28—C29—C30	121.8 (3)
C15—C14—H14	119.9	C30—C29—H29	119.1
C10—C15—C14	121.07 (19)	C25—C30—H30	118.6
C10—C15—H15	119.5	C29—C30—C25	122.8 (2)
C14—C15—H15	119.5	C29—C30—H30	118.6
			
N1—C4—C5—C6	179.70 (18)	O3—C16—C17—C18	−178.77 (17)
N1—C8—C9—C10	166.11 (15)	O3—C16—C17—C22	2.1 (3)
C1—C2—C3—C4	−0.6 (3)	O4—C16—C17—C18	1.2 (3)
C2—C1—C6—C5	−0.7 (3)	O4—C16—C17—C22	−177.96 (18)
C2—C1—C7—O1	−7.6 (3)	N2—C20—C21—C22	−179.35 (18)
C2—C1—C7—O2	172.46 (17)	N2—C23—C24—C25	−59.3 (4)
C2—C3—C4—N1	−179.11 (16)	C16—C17—C18—C19	−178.47 (18)
C2—C3—C4—C5	−0.6 (3)	C16—C17—C22—C21	177.95 (17)
C3—C4—C5—C6	1.2 (3)	C17—C18—C19—C20	0.7 (3)
C4—N1—C8—C9	−171.47 (17)	C18—C17—C22—C21	−1.2 (3)
C4—C5—C6—C1	−0.5 (3)	C18—C19—C20—N2	178.84 (19)
C6—C1—C2—C3	1.2 (3)	C18—C19—C20—C21	−1.6 (3)
C6—C1—C7—O1	172.14 (16)	C19—C20—C21—C22	1.1 (3)
C6—C1—C7—O2	−7.8 (3)	C20—N2—C23—C24	−174.9 (2)
C7—C1—C2—C3	−179.02 (15)	C20—C21—C22—C17	0.3 (3)
C7—C1—C6—C5	179.53 (18)	C22—C17—C18—C19	0.7 (3)
C8—N1—C4—C3	−16.3 (3)	C23—N2—C20—C19	−170.4 (2)
C8—N1—C4—C5	165.28 (18)	C23—N2—C20—C21	10.0 (3)
C8—C9—C10—C11	−71.6 (2)	C23—C24—C25—C26	134.2 (3)
C8—C9—C10—C15	112.22 (19)	C23—C24—C25—C30	−49.0 (4)
C9—C10—C11—C12	−175.79 (17)	C24—C25—C26—C27	175.8 (2)
C9—C10—C15—C14	176.03 (17)	C24—C25—C30—C29	−175.8 (2)
C10—C11—C12—C13	−0.3 (3)	C25—C26—C27—C28	0.5 (3)
C11—C10—C15—C14	−0.3 (3)	C26—C25—C30—C29	1.1 (3)
C11—C12—C13—C14	−0.3 (3)	C26—C27—C28—C29	0.5 (4)
C12—C13—C14—C15	0.6 (3)	C27—C28—C29—C30	−0.7 (4)
C13—C14—C15—C10	−0.3 (3)	C28—C29—C30—C25	−0.1 (4)
C15—C10—C11—C12	0.6 (3)	C30—C25—C26—C27	−1.2 (3)

### 4-[(2-Phenylethyl)amino]benzoic acid Hydrogen-bond geometry (Å, º)

**Table:**

*D*—H···*A*	*D*—H	H···*A*	*D*···*A*	*D*—H···*A*
O1—H1···O4	1.05 (4)	1.58 (4)	2.6380 (19)	177 (3)
O3—H3*A*···O2	0.82	1.81	2.6246 (18)	175

## References

[bb1] Dolomanov, O. V., Bourhis, L. J., Gildea, R. J., Howard, J. A. K. & Puschmann, H. (2009). *J. Appl. Cryst.* **42**, 339–341.

[bb3] Liu, C. & Long, S. (2023). *IUCrData*, **8**, x230599. 10.1107/S2414314623005990 PMC10626613 37937126

[bb4] Rigaku OD (2022). *CrysAlis PRO*. Rigaku Oxford Diffraction, Yarnton, England.

[bb5] Sheldrick, G. M. (2015*a*). *Acta Cryst.* A**71**, 3–8.

[bb8] Sheldrick, G. M. (2015*b*). *Acta Cryst.* C**71**, 3–8.

[bb9] Sohail, R., Mathew, M., Patel, K. K., Reddy, S. A., Haider, Z., Naria, M., Habib, A., Abdin, Z. U., Chaudhry, W. R. & Akbar, A. (2023). *Cureus*, **15**, e37080. 10.7759/cureus.37080 PMC10156439 37153279

